# Application of Vibrational Spectroscopy Supported by Theoretical Calculations in Identification of Amorphous and Crystalline Forms of Cefuroxime Axetil

**DOI:** 10.1155/2015/921049

**Published:** 2015-01-11

**Authors:** Alicja Talaczyńska, Kornelia Lewandowska, Anna Jelińska, Piotr Garbacki, Agnieszka Podborska, Przemysław Zalewski, Irena Oszczapowicz, Adam Sikora, Maciej Kozak, Judyta Cielecka-Piontek

**Affiliations:** ^1^Department of Pharmaceutical Chemistry, Faculty of Pharmacy, Poznan University of Medical Sciences, Grunwaldzka 6, 60-780 Poznan, Poland; ^2^Department of Molecular Crystals, Institute of Molecular Physics, Polish Academy of Sciences, Smoluchowskiego 17, 60-179 Poznan, Poland; ^3^Faculty of Non-Ferrous Metals, AGH University of Science and Technology, Aleja A. Mickiewicza 30, 30-059 Krakow, Poland; ^4^Department of Modified Antibiotics, Institute of Biotechnology and Antibiotics, Starościńska 5, 02-516 Warszawa, Poland; ^5^Department of Macromolecular Physics, Adam Mickiewicz University, Umultowska 85, 61-614 Poznan, Poland

## Abstract

FT-IR and Raman scattering spectra of cefuroxime axetil were proposed for identification studies of its crystalline and amorphous forms. An analysis of experimental spectra was supported by quantum-chemical calculations performed with the use of B3LYP functional and 6-31G(d,p) as a basis set. The geometric structure of a cefuroxime axetil molecule, HOMO and LUMO orbitals, and molecular electrostatic potential were also determined by using DFT (density functional theory). The benefits of applying FT-IR and Raman scattering spectroscopy for characterization of drug subjected to degradation were discussed.

## 1. Introduction 

**Figure 1:**
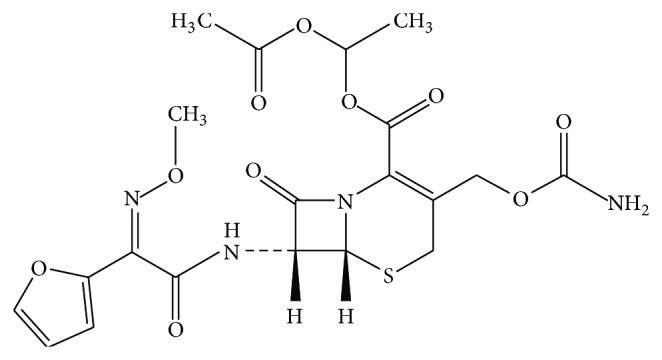
Chemical structure of cefuroxime axetil.

Cefuroxime axetil belongs to the second generation of cephalosporins characterized by a broad spectrum of antibacterial activity and is used as an oral prodrug [1]. Cefuroxime axetil contains various isomeric centers and occurs in crystalline and amorphous forms. The stereochemical properties of cefuroxime axetil are essential for its antibacterial activity and affinity to biological membranes. A* syn*-geometric isomer demonstrates considerable resistance to *β*-lactamases and is easily transported while an anti-isomer of cefuroxime axetil is deactivated by cephalosporinases [2, 3]. Due to the poor solubility of cefuroxime axetil content, studies of polymorphs in drug products are required. The abbreviated new drug application (ANDA) has approved cefuroxime axetil as an amorphous dispersion or an amorphous/crystalline mixture following the expiry of patent protection and the appearance of generic products. It is suggested that the crystalline content in the mixture of cefuroxime axetil may “seed” crystallization of the amorphous form and so increase the content of the crystalline form that is known to be less soluble and bioavailable [4]. The literature does not provide reports on analytical methods allowing precise and valid characterization of polymorphic forms of cefuroxime axetil. The methods that have been proposed, including those recommended by USP, employ polarizing microscopy [5]. They fail to supply the data necessary to study the polymorphism of cefuroxime axetil during the manufacture of batch-to-batch drug products as well as their control throughout drug product shelf-life. The development of an analytical method for examining cefuroxime axetil polymorphism needs to take into consideration the susceptibility of the drug to degradation [6, 7]. The stability of cefuroxime axetil has been studied, including evaluation of differences in kinetic degradation, adsorption, and photoisomerisation of diastereoisomers [8–10]. It is possible to suggest that interring stresses are one of the reasons of instability of *β*-lactam analogs (Figure 1).

The current work was aimed at the application of FT-IR and Raman spectroscopies for polymorphs of cefuroxime axetil. The optimized geometry, the frontier molecular orbitals, and the molecular electrostatic potential of the drug were also established.

## 2. Experimental

### 2.1. Substances for Studies

The cefuroxime axetil polymorphs were provided by the Institute of Biotechnology and Antibiotics in Warsaw, Poland. The purity of crystallic form of CA was 98.6%, content of water was 0.7%, and content of main impurity was 0.04%. The purity of amorphous form of CA was 96.5%, content of water was 0.6%, and content of main impurity was 0.07%. The particular procedure of obtaining amorphous and crystalline form was presented in another manuscript [11].

### 2.2. Vibration Spectroscopy

Vibrational infrared spectra of cefuroxime axetil were recorded between 100 and 4000 cm^−1^ in powder, at room temperature, with an FT-IR Bruker Equinox 55 spectrometer (Bruker, Billerica, MA, USA) equipped with a Bruker Hyperion 1000 microscope (Bruker, Billerica, MA, USA). Raman scattering spectra were obtained with a LabRAM HR800 spectrometer (HORIBA Jobin Yvon, Villeneuve-d'Ascq, France) with the laser excitation *λ*_exc_ = 633 nm (He-Ne laser). In each case, the power of the laser beam at the sample was less than 1 mW to avoid sample damage.

### 2.3. Scanning Electron Microscopy (SEM)

Prior to the study, all samples were coated with mixture of gold and palladium in Polaron Range SC7620 Sputter Coater. Time of coating was set to 135 seconds. The surfaces of crystalline and amorphous forms of CA were observed with the use of Hitachi S-3000N Scanning Electron Microscope (Hitachi, Japan).

### 2.4. Differential Scanning Calorimetry (DSC)

DSC analysis of crystalline and amorphous forms of CA was performed by using DSC 1 Star System (Mettler Toledo, Zurich, Switzerland). The samples were sealed in aluminum cells with pierced lids. The measurements were performed in nitrogen atmosphere within the temperature range from 40°C to 340°C, with a heating step 10°C/min. The cell constant calibration method was applied to the analysis of the DSC patterns.

### 2.5. X-Ray Powder Diffraction (XRPD)

X-ray powder diffraction studies of crystalline and amorphous forms of CA were carried out by the use of modified HZG-4 powder diffractometer using the CuK*α* radiation. The powdered samples subjected to X-ray diffraction analysis were placed on vertical polycarbonate holders. X-ray diffraction data were collected in the 2*θ* = 4–60° range at the scanning rate 0.02° s^−1^ in room temperature. The diffraction data were processed and analyzed using Origin software.

### 2.6. Theoretical Analysis

The optimization of the molecular geometry (MG), spatial electron distribution of frontier molecular orbitals (FMOs), and molecular electrostatic potential (MEP) of cefuroxime axetil were obtained with density functional theory calculations using Becke's three-parameter hybrid functional (B3LYP) implemented with the standard 6-31(d,p) as a basis set. The harmonic vibrational frequencies of FT-IR and Raman spectra were calculated using the same level of theory. All the calculations were made by using a Gaussian 03 package and a GaussView application was utilized to present the MG, FMO, and MEP [12].

## 3. Results and Discussion

**Figure 2:**
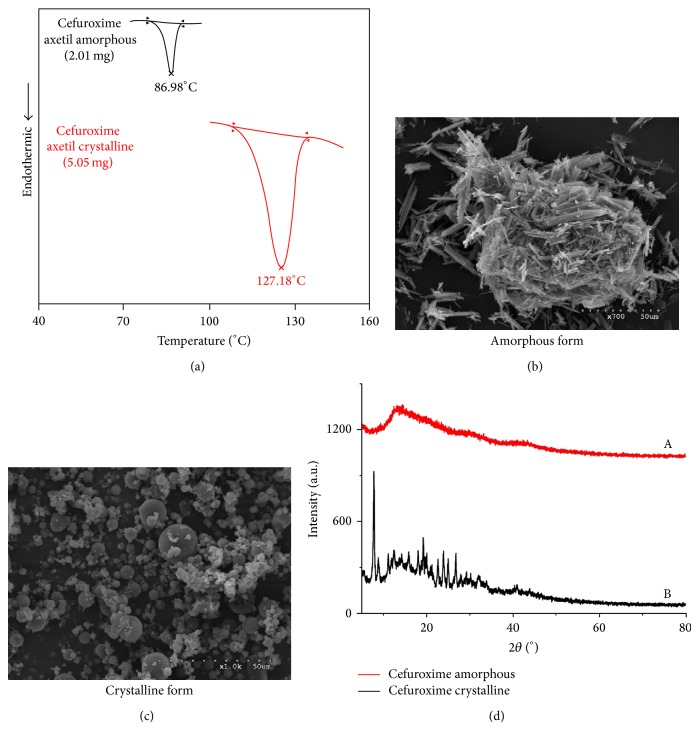
DSC curves (a), SEM images (b, c), and XRPD (d) spectra of amorphous and crystalline forms of cefuroxime axetil.

**Figure 3:**
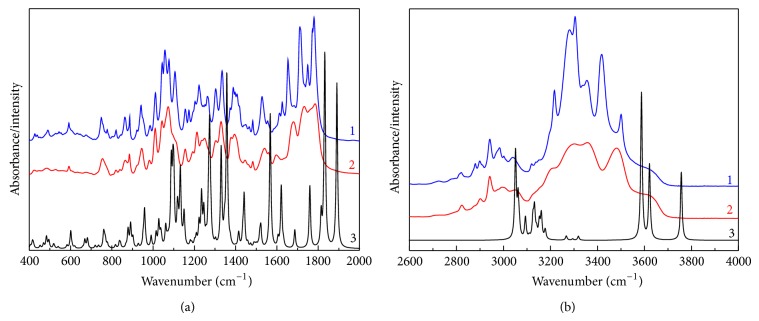
IR absorption spectra of cefuroxime axetil at room temperature, in polycrystalline powder: (1) crystalline form and (2) amorphous form. The corresponding calculated IR absorption spectra (B3LYP/6-31G(d,p)) were added for comparison (3).

**Figure 4:**
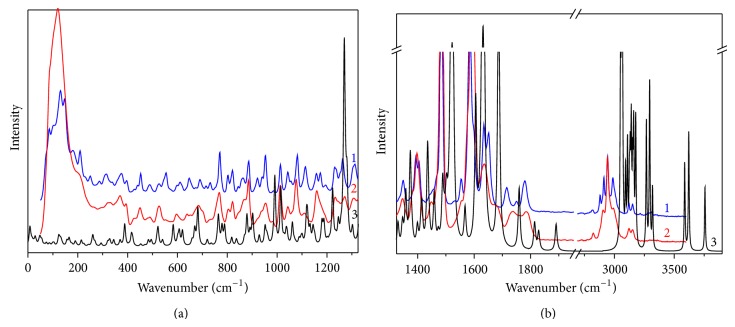
Raman scattering spectra of cefuroxime axetil at room temperature, in polycrystalline powder: (1) crystalline form and (2) amorphous form. The corresponding calculated IR absorption spectra (B3LYP/6-31G(d,p)) were added for comparison (3).

**Figure 5:**
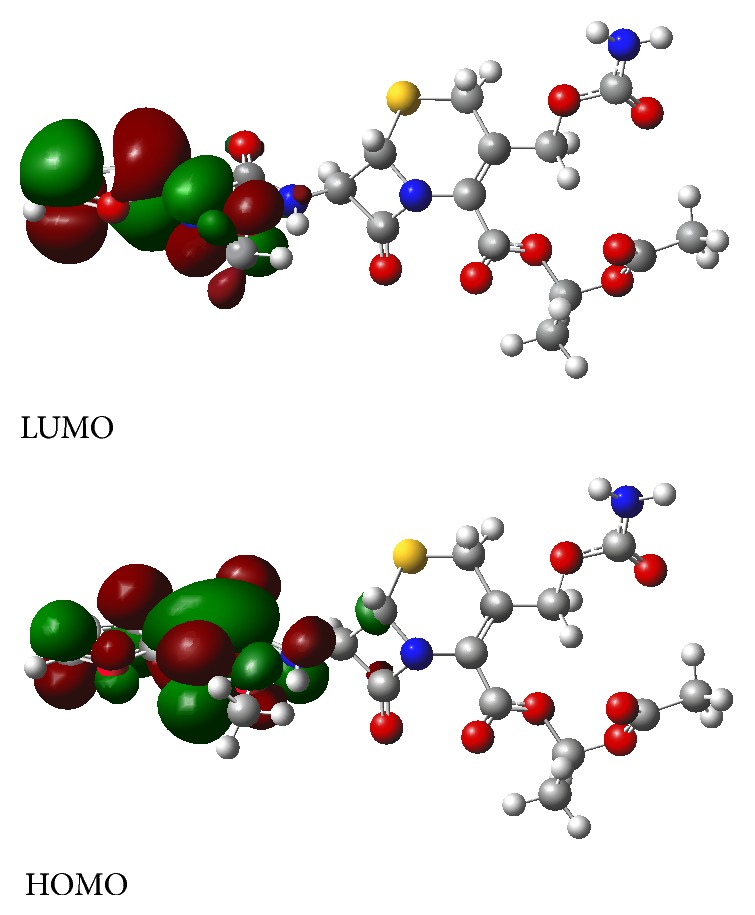
Frontier molecular orbitals of cefuroxime axetil.

The following techniques are considered for the usage in studies of polymorphism of drugs: crystallography, spectroscopy, microscopy, and thermal analysis. They permit examining different aspects of structure, dynamics, and energetics in solid state of drugs. In our investigations, we confirmed the application of vibrational spectroscopy in analysis of amorphous and crystalline forms of CA while, as reference methods, DSC, SEM, and XRD were used. The main characteristic vibrations of cefuroxime axetil were identified by comparing its experimental FT-IR and Raman scattering spectra with the theoretical ones based on the density functional theory (Table 1). The following methods, SEM, DSC, and XRPD, were used in order to confirm the presence of amorphous and crystallic forms of cefuroxime axetil. DSC, SEM, and XRPD were proposed for identification of crystalline and amorphous forms of CA (Figure 2). In DSC analysis, the crystalline form of CA exhibited single peak at 127.18°C, corresponding to its melting point. On the other hand, the amorphous form exhibited an endothermic transition with peak at 86.98°C (Figure 3). As shown in Figure 4, the characteristic hexagonal-type crystals were observed in samples of crystalline form of CA. On the contrary, morphologic analysis of amorphous form of CA indicated the presence of uniformly distributed spherical shaped aggregates. The X-ray diffraction data, showing the differences between amorphous and crystallic forms of CA, are presented in Figure 5.

The experimental and calculated spectra consistently expressed not only the energy distribution of the vibrational modes but also their relative intensities. Based on the fact that it was possible to obtain a reliable assignment of most of the observed bands to the normal modes of the cefuroxime axetil molecule during experimental studies, the shifts in band positions and the disappearance of some bands in the theoretical spectra resulted from the fact that the calculations were made for isolated molecules within the harmonic approximation, while real molecules are not isolated and vibrate anharmonically. Therefore, the calculated spectra at 836 cm^−1^ displayed a band related to the stretching vibration of the C–S bond in the 5-thia-1-azobicyclic structure and the stretching vibration of the C–C bond between the 5-thia-1-azobicyclic structure and the ((aminocarbonyl)oxy)methyl group and the bending vibration of the C–C–C bond in the furanyl ring and in the *β*-lactam ring, which had additional components related to the breathing bands in the *β*-lactam ring. Those bonds in the experimental spectra were observed at 821 cm^−1^. In the calculated spectra, distinct bands were observed at 990, 1027, and 1330 cm^−1^. These bands, primarily associated with the stretching vibration of the C–C bond in the 5-thia-1-azobicyclic structure, are related to the additional normal modes too. Thus, the band at 990 cm^−1^ was characteristic of the rocking vibration of the NH_2_ and CH_2_ groups in the ((aminocarbonyl)oxy)methyl group. A band was registered at 1027 cm^−1^ associated with the rocking vibration of the CH_2_ and amine groups in (acetyloxy)ethyl substituent and with the stretching vibration of the C–O bond in this group. The band at 1330 cm^−1^ had an additional mode related to the stretching vibration of the C–N bond in the *β*-lactam ring and the stretching vibration of the C–C bond between the 5-thia-1-azobicyclic structure and the wagging vibration of the CH_2_ in that structure and in the ((aminocarbonyl)oxy)methyl substituent. In the experimental IR spectra, these bands (except 990 cm^−1^) were also fairly intensive, very well separated, and located at 984 and 1302 cm^−1^. The band corresponding to the stretching vibration of the C–C and the scissoring vibration of the CH_2_ bonds in the furanyl(methoxyimino)acetyl group and the stretching vibration of the N–O and C–N bonds in the group between the furanyl and the 5-thia-1-azobicyclic structures was observed in the calculated spectra at 1061 cm^−1^, whereas, in the experimental IR spectra, it was located at 1012 cm^−1^. The stretching vibration of the N–O bond in the group between the furanyl and the 5-thia-1-azobicyclic structures was found in the experimental IR spectra at the 886 cm^−1^ as compared to 891 cm^−1^ in the calculated spectra.

This band probably contained additional components related to the stretching vibrations of the C–C bonds in the (acetyloxy)ethyl group and in the group between the furanyl group and the 5-thia-1-azobicyclic structure and the bending vibration of the C–C–C bond in the furanyl ring. Bands related to the stretching vibration of the C–O bonds in the (acetyloxy)ethyl group were noted in the calculated/experimental spectra at 958/940, 1132/1104, and 1274/1264 cm^−1^. The first and second bands were characteristic of the vibration of the C–O bond in the (acetyloxy)ethyl group. The third band was associated with the vibration of the C–O bonds between the first CH_3_ group and the next oxygen atom and with the stretching vibration of the C–O bond in this group. The stretching vibration of the C–O bond between the furanyl and the 5-thia-1-azobicyclic structures was located in the calculated spectra at 1088 cm^−1^, while in the experimental was at 1044 cm^−1^. The stretching vibration of the C–O bonds in the amide group was observed at 1096 and 1357 cm^−1^ in the calculated spectra and at 1055 and 1335 cm^−1^ in the experimental spectra. The first band had additional components responsible for the stretching vibration of the C–N bond in the (methoxyimino)acetyl group and the bending vibration of the C–O–N bond in the 5-thia-1-azobicyclic structure. The second was related also to the stretching vibration of the C–N bond in this group, and the wagging vibration of the CH_2_ group in the (methoxyimino)acetyl was detected as well. Four distinct bands corresponding to the stretching vibration of the C=O bond between the furanyl and the 5-thia-1-azobicyclic structures, the (acetyloxy)ethyl amide group, and the 5-thia-1-azobicyclic structure were located in the calculated IR spectra at 1759, 1814, 1832, and 1890 cm^−1^. In the experimental IR spectra, they appeared at 1709, 1749, 1772, and 1779 cm^−1^. The bands associated with the stretching vibration of the C=C bond in the furanyl group and the 5-thia-1-azobicyclic structure were observed in the calculated spectra at 1522 cm^−1^ and 1567 cm^−1^, respectively. The first band had additional components connected with the stretching vibration of the C–C bond between the furanyl and the NOCH_3_ groups in the (methoxyimino)acetyl group and with the rocking vibration of the N–H bond and the scissoring vibration of the CH_2_ and CH_3_ groups in this group. Quite strong bands corresponding to the stretching vibration of the C=N bond and the wagging vibration of the N–H bond in the group between the furanyl and the 5-thia-1-azobicyclic structure were observed in the calculated/experimental spectra at 1567/1527 cm^−1^. The bands related to the stretching of the N–H bond in the (methoxyimino)acetyl and ((aminocarbonyl)oxy)methyl groups were observed in the calculated spectra at the 3587, 3621, and 3756 cm^−1^.

Experimental and calculated Raman scattering spectra of crystalline and amorphous forms of cefuroxime axetil were also obtained. Most of the bands observed in the IR absorption spectra were observed in the Raman scattering spectra, too. The highest intensity was shown by the bands located between 1200 cm^−1^ and 1800 cm^−1^ and above 3000 cm^−1^. In the first region, bands corresponding mainly to the stretching vibration of the C–O, C–N, C=C, C=O, and C=N bonds were identified (Table 1). The most intensive bands in this region were related to the stretching vibration of the C=C and C=N bonds in the furanyl ring and they were observed in the experimental spectra at 1484 and 1584 cm^−1^. In the calculated Raman scattering spectra, they appeared at 1522 and 1631 cm^−1^, respectively. The second region was dominated by the bands associated with the stretching vibration of the C–H and N–H bonds.

The next stage of this study involved determining differences in FT-IR and Raman spectra of cefuroxime axetil that might indicate the presence of crystalline and/or amorphous forms of the drug. The amorphous and crystalline forms of cefuroxime axetil demonstrated large differences in their spectra. The majority of the absorption bands appeared at nearly the same wavenumbers and corresponded to the same vibrational mode. Significant differences in the positions, intensities, and forms of selected bands of the crystalline form of cefuroxime axetil were observed in comparison to the corresponding bands of the amorphous form. The most noticeable differences were seen in the free region in the IR absorption spectra and four regions in the Raman scattering spectra (Figures 1 and 3). In the first region of the IR absorption spectra, polymorphic differences occurred in the range 1000–1300 cm^−1^, where the bands corresponding to the stretching vibration of the C–O and C–C bonds in the furanyl ring as well as to the (methoxyimino)acetyl and (acetylo)ethyl groups were mainly observed. In this region, the disappearance of the bands in the amorphous form in cefuroxime axetil and extension of these bands. Similar changes were visible in the range 1600–1800 cm^−1^, where the bands associated with the stretching vibration of the C=N, C=O, C–H, and C–N bonds, present in the outer part of the molecule, were noticed. A very significant change was detected in the range 3000–3500 cm^−1^, where the bands associated with the stretching vibration of the N–H bonds emerged. In this region, in the IR absorption spectra, the bands corresponding to the stretching vibration of the O–H bands were also observed. An examination of the crystalline form of cefuroxime axetil demonstrated the appearance of additional bands at 3420 and 3501 cm^−1^, which was possible due to the formation of hydrogen bonds and have been reported for this form [13].

A comparison of Raman spectra obtained for the crystalline and amorphous forms of cefuroxime axetil provided important findings proving that Raman scattering spectroscopy is a powerful tool for investigation of drug polymorphism because of its remarkable sensitivity to the crystalline structure of molecular compounds. The sensitivity of the method applied in this study was greatly enhanced especially in the range of low wavenumbers, particularly below 200 cm^−1^, where Raman active vibrational modes associated with molecular skeleton deformations, librations, and translations are known to occur and which were also observed for cefuroxime axetil in this work (Figure 4). The value of energy gap is low which is connected to significant instability of *β*-lactam antibiotics.

In the Raman scattering spectra, changes in crystalline and amorphous forms were also found in other regions but they were not as intensive as those registered at low frequencies. No bands in frequencies below 200 cm^−1^ in the calculated Raman scattering spectra were observed because DFT calculations for a single molecule were not able to reproduce the lattice vibration and the calculated bands were only associated with the deformation of the molecular skeleton, which proved that polymorphic differences were detectable in this range. The calculated bands exhibited only a slight correspondence to the experimental spectrum of the amorphous form. The bands that failed to be described by DFT calculations were in the low energy region (below 100 cm^−1^) and could be associated with the translational and vibrational modes characteristic of a crystalline structure that is impossible to predict during calculations for a single molecule (Figure 4(a)). It was shown that the number of Raman active bands for the crystalline form of cefuroxime axetil was greater than that for its amorphous form. The new bands, which were not predicted by DFT calculations, might be associated with the increase of the molecules per unit cell or with the activation of infrared active modes due to the loss of the inversion center. Differences between theory and experiment were observed, which are caused by approximation of calculating FT-IR and Raman spectra for separate molecule.

The most important frontier molecular orbitals (FMOs) such as the highest occupied molecular orbital (HOMO) and the lowest unoccupied molecular orbital (LUMO) play a crucial role in the chemical stability of a molecule and in the interactions between atoms in molecules. The HOMO represents the ability of a molecule to donate an electron, while the LUMO is an electron acceptor. In the case of cefuroxime axetil, the HOMO and LUMO orbitals were mainly localized over the 2-furanyl(methoxyimino)acetyl group and the energy difference between the HOMO and LUMO orbitals, called an energy gap, was 4.1298 eV (Figure 5). The value of energy gap is low which is connected to significant instability of *β*-lactam antibiotics.

**Figure 6:**
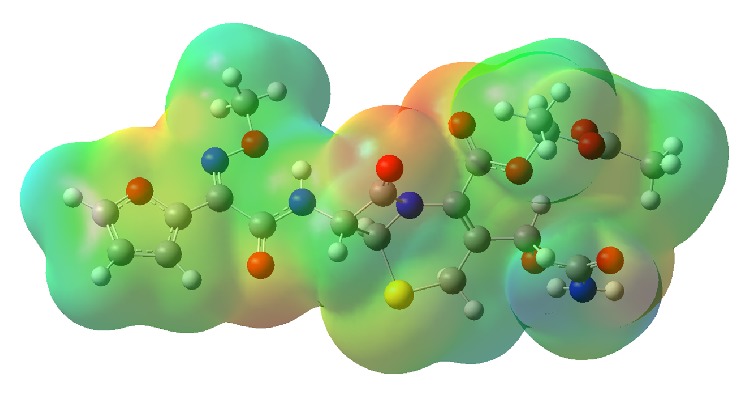
The molecular electrostatic potential of cefuroxime axetil. The positive (blue) regions of the MEP are related to electrophilic reactivity and the negative (red) to nucleophilic reactivity.

For investigating the chemical reactivity of a molecule, the molecular electrostatic potential (MEP) surface of cefuroxime axetil was plotted over an optimized electronic structure by using a density functional B3LUP method with 6-31G(d,p) as a basis set. The MEP is especially important for identification of the reactive sites for a nucleophilic and electrophilic attack in hydrogen-bonding interactions and for understanding the process of biological recognition. The different values of the molecular electrostatic potential at the surface of cefuroxime axetil are marked with different colors. The color code of the map represents the range from −5.75 10^−2 ^eV (deepest red) to 5.75 10^2^ eV (deepest blue). The positive (blue) regions of the MEP are related to electrophilic reactivity and the negative (red) to nucleophilic reactivity as shown in Figure 6. The maxima of the negative regions are localized on the (acetyloxy)ethyl ester substituent and the carbonyl group coupling with the 5-thia-1-azobicyclic structure, while the maximum of the positive region is localized on the (aminocarbonyl)oxy methyl group in cefuroxime axetil.

## 4. Conclusions

Based on the findings of this spectroscopic study, it is possible to recommend the methods applied as suitable for identification of polymorphic varieties of cefuroxime axetil. The polymorphism of cefuroxime axetil results in major changes in the vibration modes observed in the experimental and calculated IR absorption and Raman scattering spectra. The differences between the positions, intensities, and forms of certain bands of crystalline and amorphous varieties of cefuroxime axetil were more distinct in the whole range of the IR spectra whereas in the Raman spectra were mainly below 200 cm^−1^. The DFT experimental data calculations proved the presence of intermolecular interactions characteristic of crystalline forms of cefuroxime axetil.

The benefits of the techniques used in this work include method specificity, short analysis time, and, what is especially important when examining thermolabile drugs, the lack of a damaging impact on the substance studied. Therefore, the methods presented in this paper may be used to detect polymorphs during the production and storage of cefuroxime axetil.

## Figures and Tables

**Table 1:** Comparison of the observed and calculated vibrational modes of polymorphs of cefuroxime axetil.

Calculation (cm^−1^)	Experimental (cm^−1^)				Band assignment
	IR		Raman		
	Cryst.	Amorp.	Cryst.	Amorp.	
836	821	820	819	822	C–S *s* in 5-thia-1-azobicyclic structure + C–C *s* between 5-thia-1-azobicyclic structure and ((aminocarbonyl) oxy)methyl group + C–C–C *b* in furanyl ring and in β-lactam ring
878	863	864	863	863	CH_3_ *w* and O–C–O *b* in (acetyloxy)ethyl group + N–O *s* in methoxyimino group + *def*. modes in furanyl ring
891	886	885	886	883	N–O *s* and C–C *s* in (methoxyimino)acetyl group + C–C–C *b* in furanyl ring + C–O *s* in (acetyloxy)ethyl group + C–C *s* in β-lactam ring
958	940	943	940	936	C–O *s* and C–C *s* in (acetyloxy)ethyl group
990	950	—	953	952	C–C *s* in 5-thia-1-azobicyclic structure + CH_2_ *r* and NH_2_ *r* in ((aminocarbonyl) oxy)methyl group
1027	984	982	—	—	*def*. in 5-thia-1-azobicyclic structure (C–C *s*) + CH_2_ and C–O *s r* in (acetyloxy)ethyl group and for amide group in furanyl (methoxyimino) acetyl group
1061	1012	1010	1012	1012	C–C *s* and C–H *sc* in furanyl ring + N–O *s* and C–N *s* in furanyl (methoxyimino)acetyl group
1088	1044	1043	—	—	C–O *s* in OCH_3_ in furanyl (methoxyimino)acetyl group
1096	1055	—	1056	—	C–O *s* and C–N *s* in ((aminocarbonyl) oxy)methyl group + O–C=N *b* in 5-thia-1-azobicyclic structure
1118	1077	1073	1080	1076	NH_2_ *r* in ((aminocarbonyl) oxy)methyl group
1132	1104	1111	1113	1111	C–O *s*, C–H *s*,and O in (acetyloxy)ethyl group
1234	1223	1228	1232	1230	C–H *w *
1274	1264	—	1265	1269	C–O *s* in (acetyloxy)ethyl group between O and C and C–H *w* in ((aminocarbonyl) oxy)methyl group
1330	1302	1303	1303	1304	C–N *s* and C–C *s* in 5-thia-1-azobicyclic structure + C–C *s* between 5-thia-1-azobicyclic structure and ((aminocarbonyl) oxy)methyl and (acetyloxy)ethyl groups + CH_2_ *w* at 5-thia-1-azobicyclic structure and in ((aminocarbonyl) oxy)methyl group
1357	1335	1330	1335	—	C–O *s*, C–N *s*, and CH_2_ *w* in furanyl (methoxyimino)acetyl group
1414	1406	1396	1404	—	CH_3_ *sc* in (acetyloxy)ethyl group
1441	1419	—	—	—	CH_2_ *w* in ((aminocarbonyl) oxy)methyl
1522	1483	1484	1484	1484	C=C *s* in furanyl ring + C–C *s*, N–H *r*, and CH_2_ *sc* (in CH_3_ group) between furanyl ring and NOCH_3_ group in (methoxyimino)acetyl group
1567	1527	1539	—	—	C=N *s* and N–H *w* in furanyl (methoxyimino)acetyl group
1621	1626	1636	—	—	NH_2_ *sc* in ((aminocarbonyl) oxy)methyl group
1631			1584	1594	C=C *s* in furanyl ring + C=N *s* in (methoxyimino)acetyl group
1686	1652	—	1651	—	C=C *s* in 5-thia-1-azobicyclic structure
1759	1709	1732	1714	—	C=O *s* in (methoxyimino)acetyl group
1814	1749	—	1749	—	C=O *s* in (acetyloxy)ethyl groups
1832	1772	—	—	—	C=O *s* in (acetyloxy)ethyl groups + C=O *s* in ((aminocarbonyl) oxy)methyl
1890	1779	1781	1779	1787	C=O *s* at 5-thia-1-azobicyclic structure
3587	3418	—	—	—	N–H *s w* (methoxyimino)acetyl group
3621	—	—	—	—	NH_2_ *s* symmetric in NH_2_ group in ((aminocarbonyl) oxy)methyl
3756	3499	3481	—	—	NH_2_ *s *antisymmetric in NH_2_ group in ((aminocarbonyl) oxy)methyl

Vibrational modes: *s*: stretching, *b*: bending, *w*: wagging, *sc*: scissoring, *r*: rocking, and *t*: twisting.
